# Semantic dementia, progressive non-fluent aphasia and their association with amyotrophic lateral sclerosis

**DOI:** 10.1136/jnnp-2016-314912

**Published:** 2017-05-29

**Authors:** Jennifer A Saxon, Jennifer M Harris, Jennifer C Thompson, Matthew Jones, Anna M T Richardson, Tobias Langheinrich, David Neary, David M A Mann, Julie S Snowden

**Affiliations:** 1 Cerebral Function Unit, Manchester Academic Health Sciences Centre, Greater Manchester Neuroscience Centre, Salford Royal NHS Foundation Trust, Salford, UK; 2 Faculty of Biology, Medicine and Health, Division of Neuroscience and Experimental Psychology, University of Manchester, Manchester, UK

**Keywords:** ALS, FRONTAL LOBE, APHASIA

## Introduction

The association between amyotrophic lateral sclerosis (ALS) and frontotemporal dementia (FTD) is well recognised, with the majority of reports describing the behavioural form of FTD (bvFTD).^1^ Semantic dementia (SD) and progressive non-fluent aphasia (PNFA) have only rarely been reported alongside ALS.

Nevertheless, there have been a number of recent reports of language abnormalities in patients with ALS, with some authors arguing that they may be even more common than behavioural/executive impairments.^2^ ^3^ Such findings raise the question of the relationship between SD and PNFA and ALS.

To date, there has been no systematic study of the relative prevalence of bvFTD, PNFA and SD in patients with and without accompanying ALS. We examined relative frequencies in a large consecutive cohort of patients with clinical forms of FTD and suspected frontotemporal lobar degeneration pathology.

## Methods

We examined our clinical databases for all patients referred to a specialist dementia clinic within a neuroscience department between 1980 and 2016, diagnosed with one of the canonical clinical syndromes of FTD: bvFTD, SD or PNFA, or mixed variants of those syndromes. Clinical classifications were in line with diagnostic criteria for these syndromes published by Neary *et al* 4 in 1998. Most patients also fulfilled recent criteria, respectively, for bvFTD, semantic variant and non-fluent variants of primary progressive aphasia, although the SD classification included patients with right-predominant temporal lobe atrophy in whom face recognition impairments preceded problems in word comprehension. Patients were included only if they had undergone a full neurological examination and comprehensive neuropsychological assessment to support the clinical diagnosis. Cases were excluded if there was diagnostic uncertainty, for example, if a comorbid condition (eg, vascular disease, mood disorder) might have affected cognition or lack of progression suggested a possible FTD phenocopy syndrome. Importantly, patients with a logopenic aphasia profile, likely attributable to Alzheimer's disease, were excluded to avoid artificially inflating the proportion of language syndromes found in FTD without ALS.

Presence or absence of ALS was determined by clinical neurological examination, supported by neurophysiological investigations (except where impracticable). Neurological examination was repeated throughout follow-up to look for emerging signs of ALS.

We examined subsequent pathological findings where available.

We used χ^2^ analyses to compare frequency of clinical phenotypes in patients with and without accompanying ALS.

## Results

Seven hundred patients fulfilled the criteria for the study, 360 men and 340 women, mean onset age 60 (SD 8.6). Of these, 89 (12.7%) had accompanying ALS. There was no significant difference in onset age (t=−1.63(694), p=0.10) between patients with (mean=61 years, SD=9.5) and without ALS (mean=60 years, SD=8.5; four missing). There was a difference in gender distribution between the groups, with a higher male-to-female ratio in the ALS-FTD group (1.8:1) than the FTD group (1:1; χ^2^=6.50, p=0.012). There were differences in clinical phenotype (table 1). BvFTD accounted for a higher proportion of patients with ALS than those without. Patients with ALS were significantly less likely than those without to be diagnosed with SD or PNFA. Mixed behavioural language syndromes did not differentiate the two groups.

**Table 1:** Clinical classification of patients with and without ALS

	FTD only (N=611)	ALS/FTD (N=89)	χ^2^	p Value
	N (%)	N (%)		
bvFTD	307 (50.2)	70 (78.7)	25.22	<0.001***
SD	90 (14.7)	3 (3.4)	8.70	0.004**
PNFA	111 (18.2)	1 (1.1)	16.79	<0.001***
bvFTD/SD	70 (11.5)	10 (11.2)	0.004	1.00
bvFTD/PNFA	31 (5.1)	5 (5.6)	0.047	1.00
bvFTD/SD/PNFA†	2 (0.3)	0 (0)	–	1.00

**p<0.01 ***p<0.001.

†Fisher’s exact statistic used as expected cell counts below 5.

ALS, amyotrophic lateral sclerosis; bvFTD, behavioural form of FTD; FTD, frontotemporal dementia; PNFA, progressive non-fluent aphasia; SD, semantic dementia.

Thirty-seven per cent of patients with ALS-FTD were referred from a regional MND clinic, 25% from general neurologists, 27% from psychiatrists and 11% from general practitioners or other physicians. In most patients (95.5%), both FTD and ALS were present at the time of referral. In four patients (4.5%) FTD was diagnosed initially and ALS detected only on follow-up. The follow-up period exceeded 3 years in 183 patients with FTD and 5 years in 80 patients.

Ninety-nine patients have subsequently undergone postmortem examination, 13 with ALS and 86 without ALS. All 13 patients with ALS showed TDP-43 type B pathology, as defined by current nomenclature.^5^ In contrast, patients without ALS showed a range of pathologies: 39 tau, 3 FUS or FTLD-ni, and 44 TDP-43 (26 TDP-A, 9 TDP-B and 9 TDP-C). Patients with PNFA showed either tau (5 cases) or TDP-A (11 cases) pathology, whereas patients with SD (9 cases) all showed TDP-C pathology.

## Discussion

The results show that the pure syndromes of SD and PNFA occur infrequently alongside ALS. There are sound neurobiological reasons why this might be so. FTD is pathologically heterogeneous^5^ with almost half of patients having TDP-43 pathology, 45% tau pathology and a small percentage with rarer pathological types. ALS, in contrast, is more homogeneous with almost all patients having specific TDP-43 type B pathology, as found in the current cohort. TDP-B pathology is seen in some patients with bvFTD but it is not typically implicated in PNFA or SD. As exemplified by the present series, PNFA is associated with either tau or TDP-A pathology, whereas SD is consistently associated with TDP-C.

Despite the rarity of pure SD or PNFA syndromes in association with ALS, language deficits, including both syntactic and semantic impairments, have been identified as a common feature of the cognitive spectrum accompanying ALS.^2^ ^3^ It is interesting in this regard that mixed behavioural/language syndromes were seen as commonly in patients with ALS as those without. Thus, language problems are not absent in ALS-FTD. Rather, they are more likely to occur in the context of the behavioural and executive syndrome of FTD than in isolation.

In this large cohort of 700 patients, 12.7% showed evidence of ALS. This figure is remarkably similar to the figure of 12.5% reported in 40 FTD cases.^6^ In that study, subclinical motor dysfunction was found in a much larger proportion, particularly PNFA and bvFTD. The implication is that substantially greater numbers of patients should develop ALS with progression of illness, consistent with the notion of a clinicopathological continuum between FTD and ALS. Interestingly, however, the majority of patients in this study showed signs of both disorders at the time of first clinic referral, and continued follow-up elicited few additional ALS cases. A continuum in which all patients with FTD were equally susceptible to ALS might be expected to produce a more equal spread of development of ALS across an extended time period.

Further study of the nature of the language changes found in ALS-FTD will be important in order to attain a greater understanding of the neuropsychological profile of this condition.
